# Non‐Canonical, Somatic‐Dependent Vertical Transmission of *Wolbachia* in an Aphid

**DOI:** 10.1111/1758-2229.70269

**Published:** 2026-03-03

**Authors:** Tomonari Nozaki, Yuuki Kobayashi, Shuji Shigenobu

**Affiliations:** ^1^ Laboratory of Evolutionary Genomics, National Institute for Basic Biology (NIBB) Okazaki Aichi Japan; ^2^ Department of Basic Biology School of Life Science, the Graduate University for Advanced Studies, SOKENDAI Okazaki Aichi Japan

**Keywords:** cedar bark aphid, endosymbiotic bacteria, maternal transmission, viviparous reproduction, *Wolbachia*

## Abstract

*Wolbachia*, a widespread endosymbiotic bacterium that infects a broad range of arthropods and nematodes, relies on vertical transmission from mother to offspring. This process often involves colonisation of the host germline, subsequent transfer to developing oocytes, and utilisation of host yolk protein transport mechanisms such as vitellogenin uptake. However, the transmission strategies employed by *Wolbachia* in viviparous insects such as aphids are poorly understood. Here, we demonstrate a non‐canonical *Wolbachia* transmission mode in the cedar bark aphid *Cinara cedri*: the somatic‐dependent vertical transmission. After confirming consistent *Wolbachia* infection in *C. cedri*, we visualised the localization of *Wolbachia*, along with the obligate symbionts *Buchnera aphidicola* and *Serratia symbiotica* . Consistent with previous reports, *Wolbachia* in *C. cedri* were predominantly observed within maternal and embryonic bacteriocytes, the specialised cells housing obligate symbionts. Notably, *Wolbachia* cells were rarely detected in germline cells or early‐stage embryos and were directly transmitted from maternal bacteriocytes to developing embryos, coinciding with obligate symbiont transfer. These results suggest that *Wolbachia* in *C. cedri* has evolved a unique “piggybacking” strategy, utilising the obligate symbiont transmission system. Our study highlights the diversity of endosymbiont maternal transmission strategies and provides new insights into the underlying molecular mechanisms of action.

## Graphical abstract

We discovered a novel somatic‐associated *Wolbachia* transmission strategy in cedar bark aphids (*Cinara cedri*), contrasting with its typical maternal vertical transmission. This unique maternally retained somatic re‐acquisition strategy—a “piggybacking” on the highly efficient obligate symbiont transmission pathway—reveals diverse endosymbiont strategies and offers insights into underlying mechanisms.

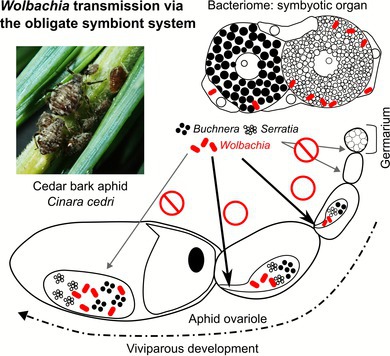

## 1 Introduction

*Wolbachia*, an alphaproteobacterial genus with widespread intracellular symbionts, infects approximately half of all arthropod and nematode species worldwide (Kaur et al. 2021). Its primary mode of transmission is vertical, from mother to offspring (Pietri et al. 2016). Maternal transmission, coupled with its remarkable ability to manipulate host reproduction, including parthenogenesis induction, cytoplasmic incompatibility, male killing, and feminization, is crucial for its evolutionary success (Kaur et al. 2021; Werren et al. 2008). Vertical transmission facilitates rapid dissemination within host populations, ensuring the long‐term persistence of *Wolbachia* (Russell et al. 2019). Although detectable in various host tissues, *Wolbachia* typically resides predominantly in the female germline (Kaur et al. 2021; Pietri et al. 2016). Although present in male germlines during early spermatogenesis, *Wolbachia* are subsequently eliminated from sperm cysts (Clark et al. 2002), effectively precluding paternal transmission. Maternal transmission is well established, occurring through *Wolbachia* accumulation within female germline stem cells and their subsequent transfer from nurse cells to developing oocytes during oogenesis (Guo et al. 2018; Hosokawa et al. 2010; Kaur et al. 2021; Russell et al. 2019).

The mechanisms of maternal transmission have been extensively studied in oviparous insects such as *Drosophila* and mosquitoes (Kaur et al. 2021; Russell et al. 2019). However, the transmission strategies employed by *Wolbachia* in viviparous insects are poorly understood. Viviparous reproduction, an alternative reproductive mode frequently observed among predominantly oviparous insects (Wheeler 2003), may present unique challenges to *Wolbachia* transmission. For instance, in the viviparous reproduction of aphids, the connection between germline cells and oocytes is limited to a brief and early developmental window, and nutrient supply from nurse cells is reasonably assumed to be restricted (Bickel et al. 2013; Miura et al. 2003). These factors may hinder the well‐known *Wolbachia* infection mechanisms. Therefore, we hypothesized that *Wolbachia* in viviparous aphids may employ one or more of the following strategies: (1) Highly efficient early germline infection with low bacterial titers, (2) co‐option of host machinery for maternal provisioning of the developing oocyte and embryo, or (3) vertical transmission through other means, such as direct transmission into the developing embryo. Detailed observations of the *Wolbachia* infection process during viviparous reproduction of aphids will expand our understanding of the diverse mechanisms of symbiont vertical transmission.

Aphids (Hemiptera: Aphididae) are prime model systems for studying intracellular symbiosis (Shigenobu and Yorimoto 2022). Almost all aphids rely on *Buchnera aphidicola* (hereafter *Buchnera*), an obligate symbiotic bacterium harboured within hyperpolyploid cells called “bacteriocytes,” for essential nutrients (Braendle et al. 2003; Nozaki and Shigenobu 2022). They frequently harbour other obligate or facultative symbiotic bacteria, such as *Serratia symbiotica* (hereafter *Serratia*) (Manzano‐Marín et al. 2023; Monnin et al. 2020; Moran et al. 2005; Yorimoto et al. 2022) in the bacteriome cells. The phylogenomic status, transmission modes, and functional roles of these symbionts have been extensively investigated (Koga et al. 2012; Oliver et al. 2010; Renoz 2024). However, the relationship between aphids and *Wolbachia* remains poorly understood largely because of the rarity of stable *Wolbachia* infections in aphids (Augustinos et al. 2011). *Wolbachia* infection has been reported in the banana aphids *Pentalonia nigronervosa* and *Pentalonia caladii* (De Clerck et al. 2014; Jones et al. 2011), and while its functional role in these species is debatable (De Clerck et al. 2015; Manzano‐Marín 2020), a recent study has shown its role in resistance to fungal parasitoids (Higashi et al. 2024). Given that *Wolbachia* strains in the M and N supergroups are primarily associated with aphids (Moreira et al. 2019; Romanov et al. 2020), a unique association between aphids and *Wolbachia* is likely. Therefore, characterising *Wolbachia* in aphids could provide valuable insights into the evolution of this highly successful insect symbiont.

*Cinara cedri*, the cedar bark aphid, is another species in which *Wolbachia* infections have been widely observed (Augustinos et al. 2011; Gómez‐Valero et al. 2004; Jousselin et al. 2016; Nozaki et al. 2022). This aphid is a well‐established model of dual‐obligate symbiosis that relies on *Buchnera* and *Serratia* for nutritional complementation (Lamelas et al. 2011; Pérez‐Brocal et al. 2006). Previous studies have shown that these symbionts, including *Wolbachia*: *Buchnera* and *Serratia*, reside in distinct bacteriocytes, and *Wolbachia* is also found within these cells rather than in other tissues or the hemolymph (Gómez‐Valero et al. 2004; Manzano‐Marín et al. 2017). However, the mode of *Wolbachia* transmission in *C. cedri* remains unclear. In the present study, we characterised the localization and transmission of *C. cedri* symbionts, including *Wolbachia*. We first confirmed consistent *Wolbachia* infection and quantified symbiont abundance using 16S bacterial rRNA amplicon sequencing in Japanese populations. We investigated the localization of the three symbionts (*Buchnera*, *Serratia*, and *Wolbachia*) within the bacteriome at different embryonic stages. Finally, based on our observations, we propose a novel *Wolbachia* transmission pathway, indicating a “piggyback” mechanism via those of obligate symbionts.

## 2 Materials and Methods

### 2.1 Aphid Collection

We collected *C. cedri* from three localities in Japan between 2021 and 2024 (Table 1). When aphids were observed on the twigs of *Cedrus deodara* , they were carefully collected using aspirators or forceps. Almost all individuals collected were viviparous in all seasons, suggesting that they were anholocyclic in Japan (but see SI text). Aphid samples were immediately preserved in 99.5% ethanol for genetic analysis after collection, or dissected and fixed with 4% paraformaldehyde (PFA) for microscopic observation. *C. cedri* has been introduced into several countries, including Japan, along with its host tree, *C. deodara* , which is grown as an ornamental plant (Blackman and Eastop 2018; Nozaki et al. 2022). We previously confirmed that Japanese colonies of *C. cedri* are genetically homogeneous based on Sanger sequencing of the cytochrome oxidase I regions (Nozaki et al. 2022).

**TABLE 1:** Sample information of *Cinara cedri* collected in the study.

#	Locality	Latitude	Longitude	Sample collection	Usage
1	Okazaki, Aichi (NIBB campus)	34.94769	137.16591	May, 2021 Jan., May 2022 Apr., Aug., Oct., Nov. 2023 May. 2024	Amplicon seq. Amplicon seq., Imaging Imaging Imaging
2	Mibu, Tochigi	36.45498	139.80784	Aug. 2022	Amplicon seq.
3	Nishinomiya, Hyogo	34.72121	135.36598	Aug. 2021	Amplicon seq.

### 2.2 16S Ribosomal DNA Amplicon Sequencing Analysis on *C. Cedri* Microbiome

To characterise the diversity and relative abundance of bacterial endosymbionts in *C. cedri*, we used 16 individuals from three geographically distinct populations across Japan (six from Aichi, six from Tochigi, and four from Hyogo; Table 1) for high‐throughput 16S rRNA sequencing targeting the hypervariable V3/V4 region of the bacterial 16S rRNA gene (Yorimoto et al. 2022). Total DNA was extracted as follows: single individuals preserved in 99.5% ethanol were air‐dried and quickly rinsed with buffer A (10 mM Tris pH 8.0, 1 mM EDTA, and 25 mM NaCl). Samples in 100 μL buffer A added to 1 μL proteinase K (400 μg/mL) were completely homogenised using BioMasher II (Nippi, Japan). After incubation at 37°C for 1 h, the samples were heated at 98°C for 2 min. The samples were preserved at −20°C until use. Using these total DNA samples, the libraries were constructed according to the 16S rRNA Sequencing Guide “16S Metagenomic Sequencing Library Preparation (15044223 B JPN),” which was provided by Illumina (CA, USA). The V3/V4 region (ca. 460 bp) of the bacterial 16S rRNA gene was amplified using the 16S AmpF_IL and 16S AmpR_IL primers. A 10 μL PCR cocktail contained 2 μL of the DNA sample, 1 μL of each primer (2 μM), 5 μL of 2× KAPA HiFi HotStart ReadyMix (KAPA Biosystems, MA, USA), and 2 μL of ultrapure water. The PCR program was as follows: 95°C for 3 min, 25 cycles at 95°C for 30 s, 55°C for 30 s, and 72°C for 30 s, and finally 72°C for 5 min. The PCR products were purified using AMPure XP beads (Beckman Coulter, CA, USA). Each PCR product was indexed using the Nextera XT Index Kit (Nextera DNA UD Indexes set B; Illunima). The quality of libraries purified with AMPure XP beads was checked using a TapeStation D1000 (Agilent, CA, USA). Pooled libraries were sequenced using the Illumina MiSeq platform (Illumina), and 250 bp paired‐end reads were generated (Table S1). Illumina raw reads were deposited in the NCBI SRA database under the accession number PRJNA1244610. Raw paired‐end reads were analysed using QIIME 2 (version 2020.8) (Bolyen et al. 2019) with the plugin “dada2” (Callahan et al. 2016) for quality filtering, trimming length, merging of paired reads, and removing chimeric sequences. Dada2‐derived amplicon sequence variants (ASVs) with < 100 reads were excluded. Amplicon sequence variants (ASVs) with high sequence identity (> 99%) were manually combined. The resultant six ASVs were manually assigned to genus‐level taxa using the BLAST function in the NCBI database. Based on the number of ASVs in individual samples, we calculated the relative abundance of each bacterium as a percentage. To infer the inter‐individual variability of symbiont abundance, the coefficient of variation (CV)—the ratio of the standard deviation to the mean—was also calculated. However, we acknowledge that 16S ribosomal DNA amplicon sequencing is subject to bias and does not provide accurate absolute abundance, mainly due to the variable number of 16S rRNA genes and bacterial genome copy numbers across species.

### 2.3 Localization of *Wolbachia* in *C. Cedri* by Fluorescence in Situ Hybridization (FISH)

To visualise the localization and vertical transmission of *Wolbachia* in *C. cedri*, we conducted FISH on bacteriome and embryos with probes that were complementary to the 16S rRNA gene sequences (W1; 5′‐Cy3‐AATCCGGCCGARCCGACCC‐3′ and W2; 5′‐Cy3‐CTTCTGTGAGTACCGTCATTATC‐3′ [Heddi et al. 1999]). The two probes were used simultaneously to increase the signal. These probes were first developed for *Wolbachia* in *Sitophilus* weevils (Heddi et al. 1999) but were later confirmed to work well in *C. cedri* (Gómez‐Valero et al. 2004). Aphids were collected three times from the campus of the National Institute for Basic Biology (NIBB) (Table 1). Fresh insects were immediately dissected in phosphate‐buffered saline (PBS; 33 mM KH_2_PO_4_, 33 mM Na_2_HPO_4_, pH 7.4) under a stereomicroscope (SZ61; Olympus, Japan) with fine forceps. We dissected the ovarioles, fat body and intestines from late‐instar nymphs or young adults of viviparous aphids to obtain a series of developing embryos. To collect bacteriomes, we dissected adults that possessed well‐developed bacteriomes. Then collected ovaries and bacteriomes were fixed in 4% PFA in PBS for approximately 3 h. The fixed samples were washed thrice with PBS‐Tx (0.3% Triton X‐100 in PBS). Samples were also washed with hybridization buffer (20 mM Tris–HCl pH 8.0, 0.9 M NaCl, 0.01% SDS, and 30% [v/v] formamide) prior to hybridization. Then samples were incubated overnight at room temperature (25°C–28°C) in hybridization buffer containing the specific probes at a final concentration of 100 nM. During overnight incubation, DNA and F‐actin were stained with 4,6‐diamidino‐2‐phenylindole (DAPI) (Dojindo, Japan) and Alexa Fluor 488 phalloidin (Thermo Fisher Scientific, MA, USA), respectively. After hybridization, the samples were washed three times in PBS‐Tx, mounted with VECTASHIELD (Vector Laboratories, CA, USA), and observed under a confocal laser scanning microscope FV1000 (Olympus). The acquired images were processed using image analysis software ImageJ (NIH, http://rsb.info.nih.gov/ij/). These observations were repeated four times.

### 2.4 Quantitative Analysis of *Wolbachia* Transmission Route

To determine the prevalence of *Wolbachia* infection across key developmental windows, we analysed the collected FISH images. Images were categorised based on the embryonic development and transmission status of the obligate symbionts (*Buchnera* and *Serratia*; details see SI text). Specifically, we quantified *Wolbachia* prevalence in the germarium (oocyte stage) and across three functional embryonic groups: stages before obligate symbiont transmission (Syncytial blastoderm and Cellular blastoderm I); stages during the transmission (Cellular blastoderm II, Invagination, and Segmentation); and stages after the transmission (Flip and Growth). The number of *Wolbachia* positive versus negative embryos/germaria was recorded for all successfully processed samples across the four independent FISH experiments. The prevalence of *Wolbachia* was then calculated for each stage (Table 2). Due to sample loss during processing, the count represents all observable structures rather than a fixed number of ovarioles per individual.

**TABLE 2:** The prevalence of *Wolbachia* and infection status of obligate symbionts in germarium and different embryonic development stages.

Stages^a^	*Wolbachia* positive embryos	*Wolbachia* negative embryos	Total *n*	*Wolbachia* prevalence (%)	Obligate symbiont transfer (*Buchnera*/*Serratia*)
Germarium	8	7	15	53.3	Prior to transfer
Early‐stage	0	38	38	0.0	Before the transmission
Mid‐stage	28	5	33	84.9	During the transmission
Late‐stage	17	0	17	100.0	After the transmission

^a^ Developmental stages were combined based on the functional status of obligate symbiont transfer. The three functional groups—early, mid, and late stages—include: the stages before *Buchnera*/*Serratia* transmission (Syncytial blastoderm and Cellular blastoderm I); the stages during the transmission (Cellular blastoderm II, Invagination, and Segmentation); and the stages after the transmission (Flip and Growth), respectively. Detailed descriptions of the developmental staging are available in the Supporting Information (Figure S3).

## 3 Results

### 3.1 Microbiome and Relative Abundance of Symbionts in *C. Cedri*

**FIGURE 1:**
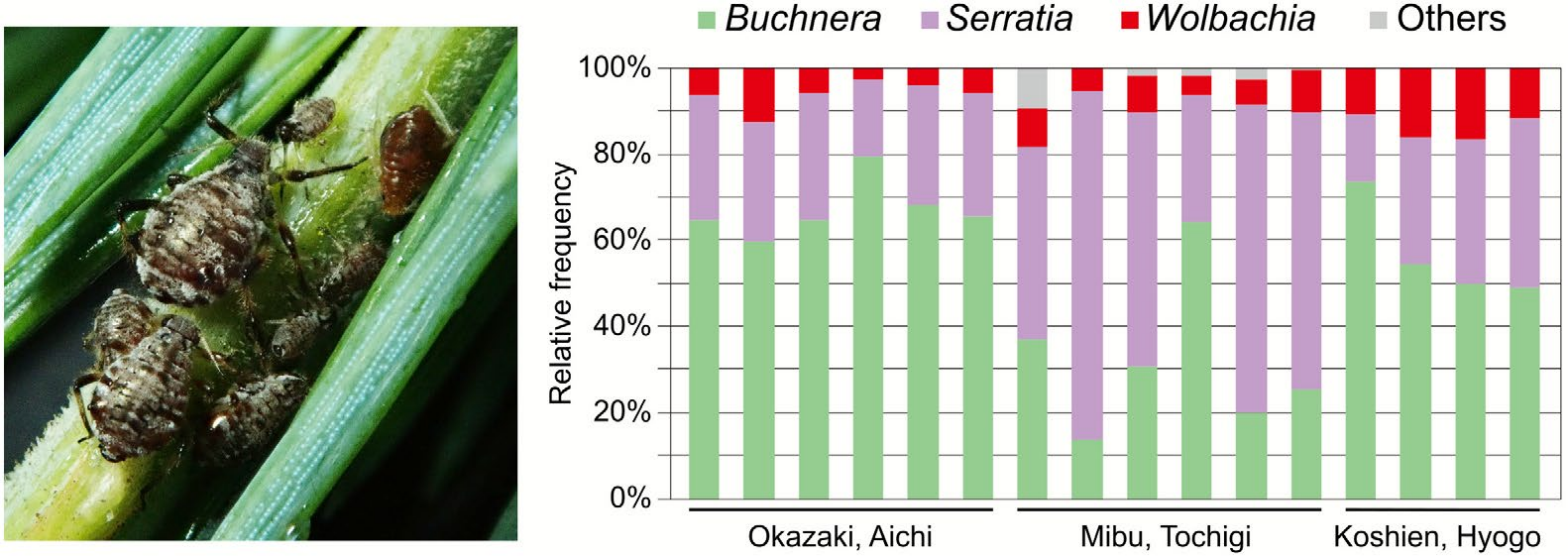
Left; image of *Cinara cedri* colony on twig of Himalayan cedar *Cedrus deodara* . Right; diversity in bacteria associated with *C. cedri*. Proportion of each bacterial species was quantified based on amplicon sequencing of hypervariable V3/V4 region of 16S rRNA gene. Assigned bacterial taxa (genus level) are colour‐coded as shown.

To quantify the bacterial diversity associated with *C. cedri*, 16 individuals collected in Japan were subjected to 16S rRNA amplicon sequencing (Figure 1, Table S1). *Wolbachia* were detected across all the populations, as well as *Buchnera* and *Serratia*, which are “obligate symbionts” in aphids (Figure 1), which was consistent with a previous report on the USA populations (Jousselin et al. 2016). To evaluate the consistency of symbiont abundance across individuals, we calculated the mean relative abundance and coefficient of variation (CV) for the three major symbionts. The relative abundance of *Buchnera*, *Serratia*, and *Wolbachia* was 51.19 ± 20.14, 39.24 ± 19.42, and 8.51 ± 4.15 (*n* = 16, mean % ± SD), respectively. The CVs of each symbiont were 0.39 (*Buchnera*), 0.50 (*Serratia*), and 0.49 (*Wolbachia*), respectively. While *Wolbachia*'s relative abundance was variable and higher than that of the primary obligate symbiont *Buchnera*, its variability is comparable to that of *Serratia*, the secondary obligate symbiont. The remaining minor bacterial taxa were not consistently detected and were revealed to be *Rosenbergiella*, *Phaseolibacter*, and *Klebsiella* allied species (Table S1).

### 3.2 Localization of *Wolbachia* Endosymbiont in *C. Cedri* Bacteriome and Embryo

**FIGURE 2:**
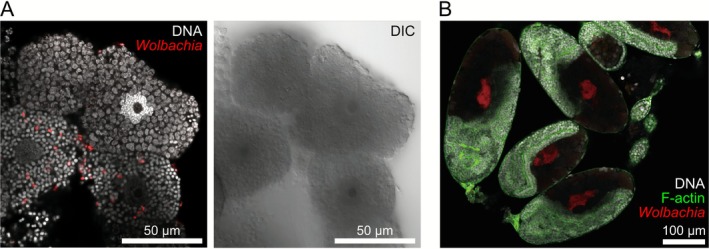
Localization of symbiotic bacteria in *Cinara cedri*. *Wolbachia* was visualised using FISH (red). (A) *Wolbachia* signals (red) were detected within bacteriocytes and sheath cells. *Wolbachia* cells were scattered throughout cytoplasm. DNA was stained with DAPI (white). (B) Signals were also detected in embryos. *Wolbachia* was recognised as a cluster within embryonic bacteriome. DNA and F‐Actin were stained with DAPI (white) and phalloidin (green), respectively.

*C. cedri* harboured three types of symbionts: *Buchnera*, *Serratia*, and *Wolbachia* (Figure 1). To determine the localization of each symbiont, we conducted FISH with specific probes targeting 16S rRNA sequences. A total of 12 adult individuals were processed for the detailed localization observation. Based on our observations of the bacteriome in adults and late instar nymphs, we determined that *Buchnera* and *Serratia* are localised in distinct bacteriocyte types: *Buchnera* and *Serratia* bacteriocytes (Figure S1). In contrast, *Wolbachia* signals were scattered throughout the cytoplasm of both the bacteriocytes and sheath cells (Figure 2A). Almost no signal was detected in intestinal or fat cells (Figure S2). This finding is consistent with a previous report (Gómez‐Valero et al. 2004). We also observed *Wolbachia* localization in developing embryos. *Wolbachia* cells were recognised as clusters in the bacteriome region of the abdominal part of the embryos (Figure 2B).

### 3.3 *Wolbachia* Vertical Transmission Through Viviparous Ovarioles in *C. Cedri*

We conducted four independent FISH experiments and successfully obtained images of 88 embryos and 15 germaria for analysis (Table 2, Table S2). To reveal the vertical transmission process of *Wolbachia*, we correlated the presence/absence of *Wolbachia* with eight embryonic stages: oocyte, syncytial blastoderm, cellular blastoderm stages I and II, invagination, segmentation, flip, and final growth (for detail, see SI text and Figures S3 and S4). Based on these categories, we first described the infection process of *Wolbachia*.

**FIGURE 3:**
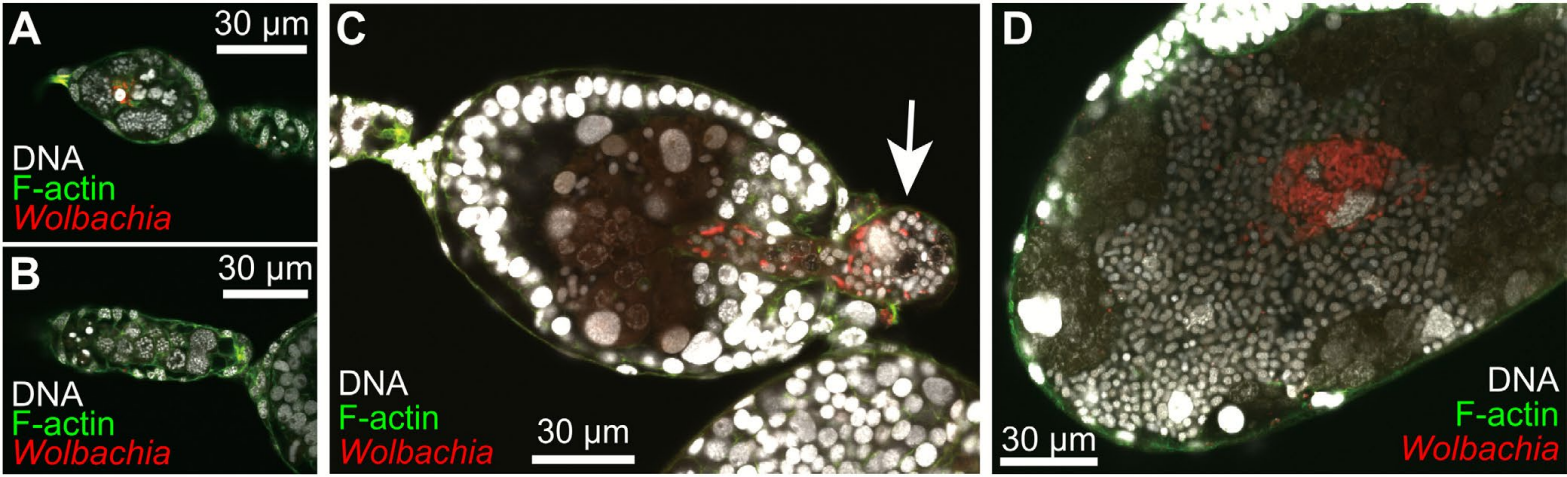
*Wolbachia* vertical transmission in *Cinara cedri*. *Wolbachia* were visualised using FISH (red), and DNA and F‐Actin were stained with DAPI (white) and phalloidin (green), respectively. (A) *Wolbachia* invaded into germ cells but their infection rates were not high. (B) *Wolbachia* cells were rarely observed in embryos at early stages, prior to transmission of main symbiotic bacteria (*Buchnera* and *Serratia*). (C) *Wolbachia*, along with obligate symbionts, were simultaneously transmitted from maternal tissues to embryo. White arrow indicates mass of symbionts at “entry point”. (D) After transmission, number of *Wolbachia* cells were noticeably increased in limited area of embryonic bacteriome.

At the oocyte stage, when the oocytes were separated from the germarium, *Wolbachia* signals were observed in the interstitial spaces of nurse cells; however, this was not consistent (germaria without *Wolbachia* signals were frequently observed) (Figure 3A). In the syncytial blastoderm and early cellular blastoderm stages, there was no signal of *Wolbachia* (Figure 3B). *Wolbachia* signals were consistently detected in the later blastoderm stage, in which both *Buchnera* and *Serratia* obligate symbionts began to be transmitted from the mother's bacteriocytes (Figure 3C). All three symbionts clustered, and the mixed population was incorporated into the embryo from the posterior region. Symbiont incorporation continued through subsequent invagination (*anatrepsis*) and segmentation stages. After the uptake of the symbionts, *Buchnera* and *Serratia* were enclosed as bacteriocytes and began to proliferate, whereas *Wolbachia* cells were scattered within the bacteriocytes or clustered in the spaces between them (Figure 3D).

Quantitatively, we then analysed the *Wolbachia* infection in terms of the core vertical transmission steps. FISH observations were grouped into four key functional stages based on obligate symbiont transfer (i.e., before, during, or after transmission) (Table 2). At the Germarium (oocyte) stage, *Wolbachia* was detected in only 53.3% of observed germaria (*n* = 15). Crucially, in the Early‐stage embryos (before obligate symbiont transmission), *Wolbachia* signals were never detected (prevalence was 0.0%, *n* = 38). The prevalence of *Wolbachia* dramatically recovered in the subsequent stages, coinciding with the maternal transmission of the obligate symbionts. In the Mid‐stage (during the transmission), *Wolbachia* prevalence reached 84.9% (*n* = 33). Finally, in the Late‐stage (after the transmission), *Wolbachia* was consistently detected in all embryos (100.0% prevalence, *n* = 17). These quantitative results show a pattern consistent with germline bypass and subsequent somatic re‐acquisition of *Wolbachia*.

## 4 Discussion

A hallmark of *Wolbachia* is its efficient and stable maternal transmission coupled with its ability to induce diverse host phenotypes, contributing to its global prevalence (Kaur et al. 2021; Pietri et al. 2016). The transmission route and its underlying mechanism are well‐studied in some *Wolbachia* infecting oviparous insects; *Wolbachia* first localise in the host maternal germline and then, transmitted to oocytes, through the connection between nurse cells and early stages of oocytes, and/or by cooption of the uptake machinery of yolk proteins (Guo et al. 2018; Russell et al. 2019; Serbus and Sullivan 2007). However, in the present study, we demonstrated that the *Wolbachia* endosymbiont in the aphid *C. cedri* utilises a non‐canonical, somatic‐associated vertical transmission route. The symbiont was directly transmitted from the maternal bacteriome to developing embryos, together with the obligate symbionts *Buchenra* and *Serratia* (Figure 3). Consistent with a previous report, *Wolbachia* in *C. cedri* was consistently detected (Figure 1; Augustinos et al. 2011; Jousselin et al. 2016; Nozaki et al. 2022) and localised in the maternal bacteriome and embryos (Figure 2; Gómez‐Valero et al. 2004). Although *Wolbachia* signals were detected in some germaria (53.3% prevalence, Table 2), their absence in the earliest stage of embryogenesis (0.0%, Table 2) indicated that transfer from germaria to oocytes did not occur (Figure 3, Table 2), precluding the canonical germline route as the primary transmission mechanism in this species.

These results suggest that *Wolbachia* in *C. cedri* piggybacked on the transmission route commonly utilised by aphid obligate symbionts and does not follow the typical strategy of this bacterial genus. Furthermore, our quantitative analysis of *Wolbachia* prevalence across developmental stages suggests a refined timing for this piggybacking. During the transmission of *Buchnera*/*Serratia*, *Wolbachia* infection was not 100% (84.9% prevalence in the Mid‐stage, Table 2). This indicated that *Wolbachia* transmission might begin after the initiation of *Buchnera*/*Serratia* transmission, resulting in a time lag compared to the highly synchronised transfer of the obligate symbionts. Considering the infection reached 100.0% in the subsequent Late‐stage embryos, the initiation timing of *Wolbachia* transmission may be more variable among individual embryos. To clarify whether this modification of the transmission pathway is caused by the reproductive specificities of the host insect or by bacterial factors, further experimental strategies are warranted. For example, comparative genomics could identify unique genes in the *C. cedri Wolbachia* strain that enable somatic targeting, while transinfection and/or curing experiments could test whether the transmission phenotype is determined by the host's reproductive physiology or the symbiont's genome.

*Wolbachia* in *C. cedri* exhibited direct transmission from maternal cells in the bacteriome to offspring with obligate symbionts (Figure 3), which is not typical of the bacterium. This deviation may be attributed to the rapid viviparous oogenesis in aphids, which is characterised by a shortened vitellogenic growth phase. Viviparous oocytes are much smaller than sexual aphids, and the connection between germarium nurse cells and oocytes is tightly restricted (Bickel et al. 2013; Miura et al. 2003). Unlike oviparous insects, there may simply not be enough time for *Wolbachia* to infect oocytes from nurse cells in the germarium. To test the hypothesis that viviparous oogenesis alone is responsible for the germline bypassing route, we therefore conducted FISH on the oviparous ovarioles in this aphid (SI text). Nevertheless, our additional observations of the oviparous ovarioles in this aphid contradicted this hypothesis; *Wolbachia* were not localised in germarium cells and were transmitted together with obligate symbionts (SI text and Figure S5). Therefore, factors other than viviparous oogenesis, such as *Wolbachia*'s ability to target and interact with diverse cellular components and organelles (Porter and Sullivan 2023), or aphid immune responses (Zug and Hammerstein 2015), warrant future genomic and experimental investigations. While our data strongly suggest the absence of *Wolbachia* in the earliest stages, future high‐resolution techniques, such as electron microscopy, are required to completely rule out an extremely low‐abundance, transient infection route.

The finding that *Wolbachia* in *C. cedri* utilises a somatic‐dependent pathway is important when considering the broader context of *Wolbachia* transmission. While the canonical germline‐to‐oocyte model is predominantly established through detailed studies in Diptera (*Drosophila* and mosquitoes) (Kaur et al. 2021; Russell et al. 2019), growing evidence reveals non‐canonical strategies in other insect taxa. For instance, in a psyllid, *Diaphorina citri* , and *Bemisia* whiteflies, *Wolbachia* exhibits an externally mediated route; that is, the bacterium is present in the egg stalk or pedicel before the egg is laid, with delayed invasion into the oocyte following oviposition (Lv et al. 2023; Ren et al. 2018). These external routes, while also bypassing the germarium, show a pronounced temporal separation from obligate symbiont transmission; the *Wolbachia* invasion often occurs after the offspring embryo or egg has already internalised the obligate symbionts (Brumin et al. 2012; Dan et al. 2017). Similarly, in an isopod, *Armadillidium vulgare* , it has been suggested that the somatic re‐infection is involved during oogenesis (Genty et al. 2014). The *C. cedri* strategy, with its maternally retained somatic re‐acquisition that is tightly coupled with the obligate symbiont system, thus expands the known spectrum of non‐canonical *Wolbachia* transmission routes. Our study underscores the diverse transmission strategies of this endosymbiont and highlights the need for more foundational, high‐resolution observations across a broader range of insect taxa.

In the present study, we exploited an introduced population of *C. cedri* in Japan (Nozaki et al. 2022) and successfully observed the transmission of symbionts in detail. In Japan, only *Wolbachia* supergroup M, an aphid‐specific clade, has been detected (Nozaki et al. 2022), whereas supergroup B has also been reported in the eastern Mediterranean, a potential native range (Augustinos et al. 2011; Michelena et al. 2005). Given the unique transmission pattern observed in this study, where the canonical germline route is highly unlikely, investigating the vertical transmission routes of other *Wolbachia* strains (e.g., supergroup B) in this aphid species is thus crucial, as it will clarify whether the observed modifications in *Wolbachia* transmission are bacterial lineage‐specific. It is also worth elucidating the transmission of the M‐supergroup strain of *Wolbachia* in banana aphids, which is the best‐studied aphid group in terms of its association with *Wolbachia* (De Clerck et al. 2014; Higashi et al. 2024; Jones et al. 2011). Additionally, future comprehensive studies quantifying the infection prevalence across various geographical populations are essential to fully determine the stability of *Wolbachia* vertical transmission, while our quantitative data from the Japanese population strongly suggest a near‐perfect maternal transmission rate in the observed individuals (Table 2).

## 5 Conclusion

In summary, we report a novel somatic‐associated *Wolbachia* transmission strategy in an aphid, *C. cedri*. Our quantitative data (0.0% prevalence in the earliest stage) demonstrate that the canonical germline route is functionally bypassed, revealing that *Wolbachia* “piggybacks” on the host machinery via the highly efficient obligate symbiont transmission pathway. This unique maternally retained somatic re‐acquisition strategy, alongside other non‐canonical examples in different insect taxa (e.g., external infection in psyllids and whiteflies), highlights the evolutionary diversity of *Wolbachia* transmission strategies. Future genomic and experimental studies, including comparative genomics among related *Wolbachia* species and artificial infection experiments, are crucial for elucidating the mechanisms driving this unique adaptation. Our findings provide an essential framework for such future studies on the molecular determinants of endosymbiont vertical transmission, including targeting/migration capabilities of *Wolbachia* (Porter and Sullivan 2023), and interactions with other symbionts and the host (Bright and Bulgheresi 2010; Vautrin and Vavre 2009; Zug and Hammerstein 2015).

## Author Contributions

**Tomonari Nozaki:** conceptualization, data curation, formal analysis, funding acquisition, investigation, methodology, visualization, project administration, resources, validation, writing – original draft, writing – review and editing. **Yuuki Kobayashi:** data curation, investigation, methodology, resources, writing – original draft, writing – review and editing. **Shuji Shigenobu:** conceptualization, funding acquisition, investigation, project administration, writing – original draft, writing – review and editing.

## Funding

This work was supported by Japan Society for the Promotion of Science, 19J01756, 22K14901, 17H03717, 17H06384, 20H00478, 25K18554.

## Ethics Statement

The insect samples used in this study were collected from the wild. The experimental methods employed do not require ethical approval.

## Conflicts of Interest

The authors declare no conflicts of interest.

## Supporting information


**Data S1:** emi470269‐sup‐0001‐Supinfo.docx.

## Data Availability

The data that support the findings of this study are openly available in NCBI at https://www.ncbi.nlm.nih.gov/, reference number PRJNA1244610.
